# Ricinus communis membrane for orbital reconstruction

**DOI:** 10.1590/S1808-86942011000200020

**Published:** 2015-10-19

**Authors:** João Daniel Caliman e Gurgel, Lydio Alves Filho, Vespasiano Lopes de Farias, André Moyses Portugal, Krishnamurti Matos de Araújo Sarmento Júnior

**Affiliations:** 1 Master's degree and doctoral student in medicine (otorhinolaryngology), Medical College of the São Paulo Holy House (Santa Casa de São Paulo). Otorhinolaryngologist and craniomaxillofacial surgeon; 2 Full member of the Brazilian Plastic Surgery Society, plastic surgery; 3 Full member of the Brazilian Plastic Surgery Society. Head of the Craniomaxillofacial Surgery Unit, Dr. Dório Silva Hospital; 4 Member of the Brazilian Otorhinolaryngology and Cervicofacial Surgery Association. Otorhinolaryngologist; 5 Master's degree in surgery, Rio de Janeiro Federal University. Otorhinolaryngologist. Rio Doce Hospital

**Keywords:** bioprosthesis, orbital fractures, traumatology

## INTRODUCTION

Blunt trauma of the periorbital area or fractures of the zygomaticomaxillary complex are common in facial trauma; these injuries often extend to the floor of the orbit.^1^ These traumas may result in major deformities, which may include injury of the ocular globe and loss of vision.^2^ Blow-out fractures of the floor of the orbit, even plain fractures, may cause functional and cosmetic disorders such as residual dystopia, limited ocular globe movement, diplopia, and enophthalmus due to herniated periorbital fat into the maxillary sinus that is often accompanied by entrapment of the extra-ocular muscle between bone fragments. Surgery for the treatment of orbital fractures, when indicated, involves reconstructing the walls of the orbit to provide support for its content during the initial healing phase; the aim is to achieve good esthetic and functional results. Several materials have been used for reconstructing the walls of the orbit, such as: titanium mesh, medpore, autogenous bone, and alloplastic bioresorbable materials.^1^

The purpose of this study was to report two cases of orbit reconstruction with a castor seed polymer membrane.

## CASE REPORTS

Patient one: a history of a fracture of the zygomaticomaxillary complex by mugging during robbery. The patient presented a right bipalpebral hematoma and edema, diplopia, malar flattening and fractures of the zygomaticomaxillary crest, the zygomaticofrontal suture, and the lower orbital margin; there was also epistaxis and a nose fracture.

Patient two: a history of a vehicle accident resulting in cranial-encephalic trauma, a subdural hematoma, a sagittal fracture of the palate, a fracture of the zygomaticomaxillary complex (zygomaticomaxillary crest, frontal ramus of the zygomatic bone, lower orbital margin, the floor and the medial wall of the orbit, ocular dystopia, avulsion of the tooth 11, and fracture of teeth 31 and 41.

**Figure 1:**
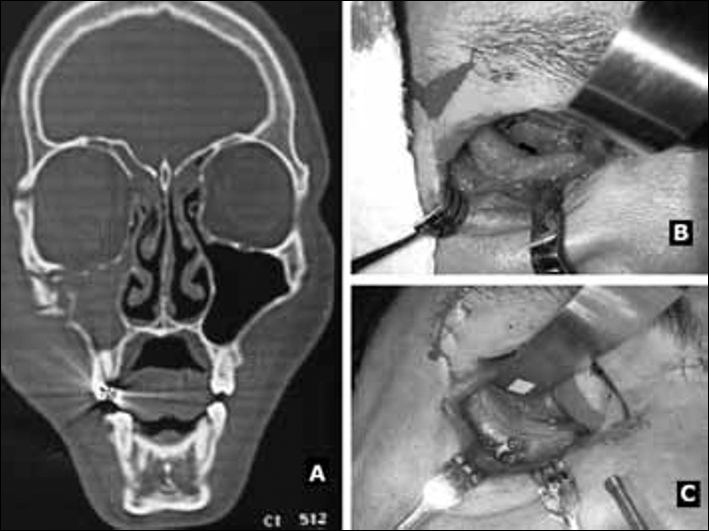
Patient one: A: Preoperative computed tomography of the facial bones showing signs of fractures on the floor of the orbit to the right, on the zygomaticomaxillary crest, and on the ipsilateral zygomaticofrontal suture area; B: Defect on the floor of the right orbit; C: Reconstructed floor using castor seed polymer membrane.

Both patients underwent surgery involving reduction of the fractures, rigid internal fixation with titanium miniplates and specific 1.3 and 1.5 mm screws, and reconstruction of the floor of the orbit (and medial wall in patient two) using a 0.5 mm thickness castor seed polymer membrane (Fig. 1). The membrane was immersed in warm saline solution and molded to the contours of the orbital walls. Patients were asymptomatic 24 months later. Diplopia and ocular dystopia resolved completely. There was no inflammation on the graft sites using the castor oil seed polymer during the follow-up period.

## DISCUSSION

There are several materials available for restoring the orbit walls; there is no consensus on which is the best. The ideal material for rebuilding the orbit - particularly the floor - should be sufficiently strong to support the content of the orbit, affordable, biocompatible, and preferably bioresorbable. The growing demand for different reconstruction materials to treat orbital fractures - restoring the margins and its volume - has had a direct impact on surgery.^2^

Studies have shown that polyurethane polymers derived from castor oil are biocompatible, non-toxic, poorly hydrophilic, and that foster regeneration and incorporation of organic tissues.^3^ Several authors have demonstrated experimentally that castor seed membranes are biocompatible and induce minor or no foreign body reaction. These researcher have also found that bone renegeration/neoformation replaces the implant, which is gradually absorbed.^4^, ^5^, ^6^

Large case series studying the castor seed polymer membrane for orbit reconstruction in human beings have not been published.

## FINAL COMMENTS

The castor seed polymer membrane is an affordable, non-toxic, well-tolerated material that is a good choice for the treatment of orbit wall fractures.
